# Effect of betaine supplementation on power performance and fatigue

**DOI:** 10.1186/1550-2783-6-7

**Published:** 2009-02-27

**Authors:** Jay R Hoffman, Nicholas A Ratamess, Jie Kang, Stefanie L Rashti, Avery D Faigenbaum

**Affiliations:** 1 Department of Health and Exercise Science, The College of New Jersey, PO Box 7718, Ewing, New Jersey 08628, USA

## Abstract

**Background:**

The purpose of this study was to examine the efficacy of 15 days of betaine supplementation on muscle endurance, power performance and rate of fatigue in active college-aged men.

**Methods:**

Twenty-four male subjects were randomly assigned to one of two groups. The first group (BET; 20.4 ± 1.3 years; height: 176.8 ± 6.6 cm; body mass: 77.8 ± 13.4 kg) consumed the supplement daily, and the second group (PL; 21.4 ± 4.7 years; height: 181.3 ± 5.9 cm; body mass: 83.3 ± 5.2 kg) consumed a placebo. Subjects were tested prior to the onset of supplementation (T1) and 7 (T2) and 14 days (T3) following supplementation. Each testing period occurred over a 2-day period. During day one of testing subjects performed a vertical jump power (VJP) and a bench press throw (BPT) power test. In addition, subjects were required to perform as many repetitions as possible with 75% of their 1-RM in both the squat and bench press exercises. Both peak and mean power was assessed on each repetition. On day two of testing subjects performed two 30-sec Wingate anaerobic power tests (WAnT), each test separated by a 5-min active rest.

**Results:**

No differences were seen at T2 or T3 in the repetitions performed to exhaustion or in the number of repetitions performed at 90% of both peak and mean power between the groups in the bench press exercise. The number of repetitions performed in the squat exercise for BET was significantly greater (p < 0.05) than that seen for PL at T2. The number of repetitions performed at 90% or greater of peak power in the squat exercise was significantly greater for BET at both T2 and T3 than PL. No differences in any power assessment (VJP, BPT, WAnT) was seen between the groups

**Conclusion:**

Two-weeks of betaine supplementation in active, college males appeared to improve muscle endurance of the squat exercise, and increase the quality of repetitions performed.

## Background

Betaine is a trimethyl derivative of the amino acid glycine. It is a significant component of many foods including wheat, spinach, beets, and shellfish [1]. It is estimated that the daily intake of betaine in the human diet ranges from an average of 1 g·d^-1 ^to a high of 2.5 g·d^-1 ^in those individuals that have a diet high in whole wheat and shellfish [2]. In addition, betaine can also be synthesized in the body through the oxidation of choline-containing compounds [2]. Some of the physiological functions attributed to betaine include acting as an osmoprotectant [3]. That is, it protects the cell against dehydration by acting as an osmolyte thereby increasing the water retention of cells. Other studies have indicated that betaine supplementation may lower plasma homocysteine concentrations [4,5] and reduce inflammation [6], providing a potential reduction in cardiovascular disease risk. In addition, betaine also acts as a methyl donor by providing a methyl group to guanidinoacetate via methionine that can synthesize creatine in skeletal muscle [7].

In consideration of these physiological effects it has been hypothesized that supplementation with betaine may have ergogenic properties (enhance sports performance) by supporting cardiovascular function or thermal homeostasis during exercise in the heat [8], and/or by enhancing strength and power performance from an increase in skeletal muscle creatine concentration [2]. Until recently, betaine has been primarily used as a dietary food supplement in animal nutrition. Studies have shown that betaine supplementation can protect fish as they move from waters of varying salinity by acting as an osmolyte [9]. In addition, betaine has been shown to enhance growth and reduce body fat in pigs [10,11], and improve recovery from exercise in untrained horses [12]. In humans, betaine has only recently been examined as a potential ergogenic aid. Armstrong and colleagues [8] examined the effect of acute betaine ingestion following a dehydration protocol and prolonged treadmill running (75 minutes at 65% of VO_2 _max) in the heat. Following the treadmill running subjects were required to perform a sprint to exhaustion. The investigators were unable to report any ergogenic benefit in regards to time to exhaustion in the sprint test. In addition, the investigators also reported a greater loss in plasma volume in subjects consuming fluids with betaine than subjects consuming fluids that did not contain betaine. They suggested that perhaps a longer supplementation period would be necessary to realize any ergogenic benefit, and that possibly the use of other modes of exercise may provide a different outcome. Subsequently, Maresh and colleagues [13] examined 14-days of betaine supplementation on strength and power performance in recreationally trained men. They found no significant changes in repetitions performed in the squat or bench press exercise, but they did find significant improvements in bench press throw power, isometric bench press force, vertical jump power and isometric squat force. Considering that this was the only study to have shown significant performance benefits from betaine supplementation in humans, additional research is warranted to confirm these results and to provide further insight to betaine supplementation. Thus, the purpose of this study was to examine the efficacy of 15 days of betaine supplementation on muscle endurance, power performance and rate of fatigue in active college-aged men.

## Methods

### Subjects

Twenty-four male subjects volunteered for this study. Following an explanation of all procedures, risks, and benefits, each subject gave his informed consent to participate in this study. The Institutional Review Board of the College of New Jersey approved the research protocol. Subjects were not permitted to use any additional nutritional supplementation and did not consume anabolic steroids or any other anabolic agents known to increase performance. Screening for steroid use and additional supplementation was accomplished via a health questionnaire filled out during subject recruitment. All subjects were recreationally active for at least the past three months including participation in a resistance training program.

Subjects were matched for size and strength and were randomly assigned to one of two groups. The first group (BET; 20.4 ± 1.3 years; height: 176.8 ± 6.6 cm; body mass: 77.8 ± 13.4 kg; body fat %: 11.6 ± 4.0%) consumed the supplement daily, and the second group (PL; 21.4 ± 4.7 years; height: 181.3 ± 5.9 cm; body mass: 83.3 ± 5.2 kg; body fat %: 12.0 ± 3.0%) consumed a placebo. The study was conducted in a double-blind format.

### Study Protocol

**Figure 1:**
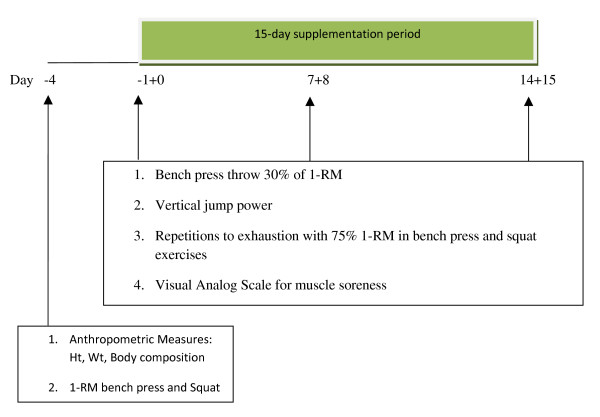
**Schematic Diagram: Testing Protocol**.

Subjects reported to the Human Performance Laboratory (HPL) on seven separate occasions. On the first visit subjects were tested for maximal strength [one repetition-maximum (1-RM)] on the squat and bench press exercises. The subsequent six visits occurred within three testing periods (T1 – T3), each separated by 7 days. Each testing period involved two days of assessment. During the first day of each testing period subjects performed a vertical jump power and an upper body (bench press throw) power test. In addition, subjects were required to perform as many repetitions as possible with 75% of their 1-RM in both the squat and bench press exercises. The two power tests were performed prior to the repetitions to exhaustion test. However, the order of the power tests and sets to exhaustion was randomly determined. Subjects returned to the HPL 24 hours later to perform two 30-sec Wingate anaerobic power tests. Each test was separated by a 5-min active rest. Following the Wingate anaerobic power test on T1 subjects began the 15 day supplement period. Subjects returned to the HPL on days 7 and 8 (T2) and days 14 and 15 (T3) to repeat the same performance tests. All tests were performed at the same time of day. Subjects also completed a Profile of Mood States and a Visual Analog Scale (VAS) for muscle soreness prior to the Wingate anaerobic power testing during each testing session. Figure 1 depicts the testing protocol.

### Maximal Strength Testing

The 1-RM tests were performed using methods previously described by Hoffman [14]. Each subject performed a warm-up set using a resistance that was approximately 40–60% of his perceived maximum, and then performed 3–4 subsequent trials to determine the 1-RM. A 3 – 5 minute rest period was provided between each trial. No bouncing was permitted for the bench press exercise, as this would have artificially boosted strength results. Bench press testing was performed in the standard supine position: the subject lowered an Olympic weightlifting bar to mid-chest level and then pressed the weight until his elbows were fully extended. The squat exercise required the subject to place an Olympic bar across the trapezius muscle at a self-selected location. Each subject descended to the parallel position which was attained when the greater trochanter of the femur reached the same level as the knee. The subject then ascended until full knee extension.

### Performance Measures: Repetitions to Exhaustion

Subjects performed one set to exhaustion on both the bench press and squat exercises. The loading for each exercise was 75% of the subjects previously determined 1-RM. Subjects were permitted to warm-up prior to the set. Subjects were instructed to perform as many repetitions as possible using proper lifting technique. Repetitions not meeting the range of motion criteria (parallel position for the squat exercise, and bar touching chest followed by full extension of the elbows for the bench press exercise) were discarded. The total number of repetitions performed was recorded.

Power output during the squat and bench press exercises was measured for each repetition with a Tendo™ Power Output Unit (Tendo Sports Machines, Trencin, Slovak Republic). The Tendo™ unit consists of a transducer attached to the end of the barbell which measured linear displacement and time. Subsequently, bar velocity was calculated and power was determined. Both peak and mean power output were recorded for each repetition and used for subsequent analysis. To determine the quality of repetitions performed, the number of repetitions performed at 90% of peak and mean power was also calculated. Test-retest reliability for the Tendo unit in our laboratory has consistently shown *R *> 0.90.

### Anaerobic Power Measures

To quantify anaerobic power performance all subjects performed a Wingate anaerobic power test (Lode Excalibur, Groningen, The Netherlands). After a warm-up period of 5 min of pedaling at 60 rpm interspersed with an three all-out sprints lasting 5 s, the subjects pedaled for 30 s at maximal speed against a constant force (1.2 Nm·kg^-1^). Subjects then performed an active rest for 5-minutes (repeat warm-up period) and then performed a second Wingate test. Peak power, mean power, total work and rate of fatigue were determined. Peak power was defined as the highest mechanical power output elicited during the test and mean power was defined as the average mechanical power during each 30-s test.

To quantify vertical jump power subjects performed a 3-jump power test. Following a brief warm-up period that included pedaling on a cycle ergometer for 5 min at 60 rpm, subjects stood on a portable force plate (Advanced Medical Technology Inc., Watertown, MA). The subject's hands were placed on his waist at all times. Upon cue each subject performed 3 consecutive vertical jumps with a standardized countermovement. The subject was instructed to maximize the height of each jump while minimizing the contact time with the force plate between jumps. Computer analysis was used to calculate power output. The highest power outputs were recorded.

To quantify upper jump power subjects performed 3-repetitions with the bench press throw exercise. All bench press throws were performed on a Cormax bench throw device (Cormax Strength Power Systems; Valley City, ND) using 30% of the subject's previously measured 1-RM bench press. The Cormax bench press throw apparatus provides a hydraulic mechanism that can unload the eccentric phase of contraction. Subjects performed all repetitions with the eccentric phase unloaded. During the concentric phase subjects were instructed to press the weight as fast as possible and release the bar as they reached the end of the range of motion. Power output during the bench press throw was measured for each repetition with the Tendo™ Power Unit as described above. Both peak power and peak force outputs were calculated and the highest outputs were recorded.

### Dietary Recall

Three-day dietary records were completed during the week prior to the onset of the study. Subjects were instructed to record as accurately as possible everything they consumed during the day and between meal and late evening snacks. FoodWorks Dietary Analysis software (McGraw Hill, New York, NY) was used to analyze dietary recalls.

### Questionnaires

The profile of mood states (POMS) was administered on the second day of each testing session. All questionnaires were performed under controlled conditions (a quiet room alone with the investigator) and the same researcher performed all test administrations. The POMS consists of 65 words or phrases in a Likert format questionnaire which provides measures of specific mood states. It provides measures of tension, depression, anger, vigor, fatigue and confusion. McNair et al., [15] has reported internal consistency of measures ranging between 0.85 to 0.95 and test-retest reliability estimates ranging between 0.65 to 0.74. These lower coefficients of stability are thought to be indicative of transient and fluctuating characteristics of mood states. During all test administrations participants were asked to describe their feelings upon how they were feeling at that moment.

Subjects were also instructed to assess their soreness of the lower body on the second day of testing using a 10 cm visual analog scale (VAS). The VAS and POMS were assessed 15 minutes prior to performance on the Wingate test. Subjects were asked to assess how they feel at that time with words anchored at each end of the VAS that expresses the most positive (no soreness) and most negative (maximum soreness) rating.

### Supplement Schedule

Subjects consumed either the supplement (1.25 g of betaine mixed in 240 ml of a sport drink) or placebo (sports drink only) twice per day. The betaine for the supplement was extracted from sugar beet molasses. Both the supplement and placebo were identical in appearance and taste. Since the betaine was added to the sports drink, the seal on the lid of each sports drink for the placebo group was also cracked to provide the same appearance as the supplement drink. Subjects consumed the first drink either in the morning or 90 min prior to the testing session, and the second drink in the evening. All drinks were consumed in the HPL, except for weekends. Subjects, following Friday's consumption were given their supplement or placebo for the weekend. Supplementation continued for 15 days. The betaine supplement was obtained from Danisco USA, Inc (New Century, KS, USA).

### Statistical Analysis

Statistical evaluation of performance changes was accomplished using 2 × 2 (time × group) analysis of variance. Statistical evaluation of dietary analysis was accomplished using unpaired t-tests. Significance was accepted at an alpha level of *p *≤ 0.05. All data are reported as mean ± SD.

## Results

Dietary recalls showed no difference between the groups in energy expenditure (2639 ± 790 kcal), total protein (143.2 ± 70.7 g), total carbohydrates (316 ± 109 g) and total fat (94.2 ± 41.7 g). The macronutrient composition of the diet for all subjects was 21.0 ± 6.7% protein, 46.6 ± 8.8% carbohydrate and 29.9 ± 7.1% fat.

**Figure 2:**
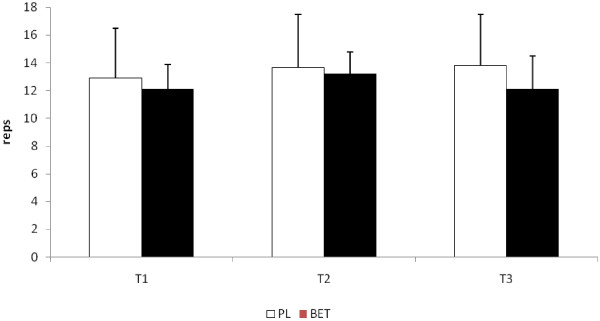
**Total Number of Repetitions Performed in the Bench Press Exercise**. Data are reported as mean ± SD. BET = Betaine; PL = Placebo.

**Figure 3:**
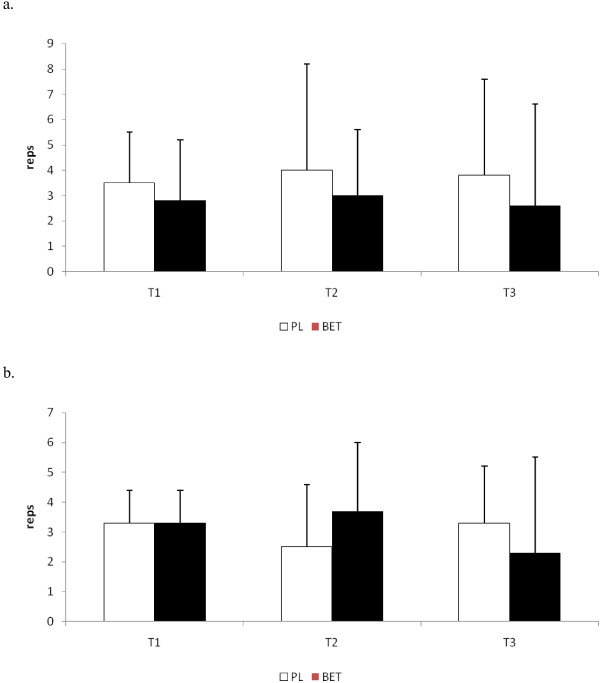
**a: Total Number of Repetitions Performed at 90% of Peak Power in the Bench Press Exercise**. **b: **Total Number of Repetitions Performed at 90% of Mean Power in the Bench Press Exercise. BET = Betaine; PL = Placebo.

**Figure 4:**
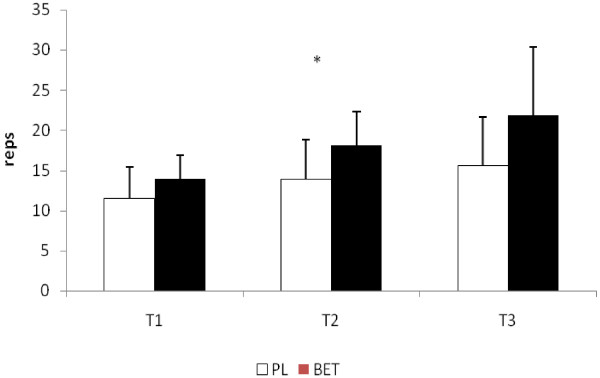
**Total Number of Repetitions Performed in the Squat Exercise**. Data are reported as mean ± SD. .* = Significantly different (p < 0.05) between BET and PL. BET = Betaine; PL = Placebo.

**Figure 5:**
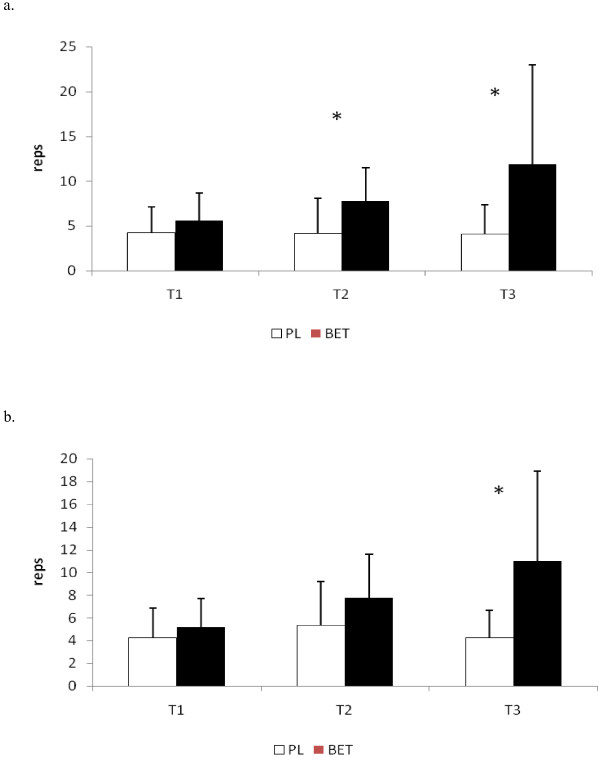
**a: Total Number of Repetitions Performed at 90% of Peak Power in the Squat Exercise**. **b: **Total Number of Repetitions Performed at 90% of Mean Power in the Squat Exercise. * = Significantly different (p < 0.05) between BET and PL. Data are reported as mean ± SD. BET = Betaine; PL = Placebo.

No significant differences were seen at T2 or T3 in the total repetitions performed to exhaustion between BET and PL in the bench press exercise (see Figure 2). In addition, no differences between the groups were seen in the number of repetitions performed at 90% of both peak and mean power at those time points (see Figure 3a and 3b, respectively). The number of repetitions performed in the squat exercise at T2 for BET was significantly greater (p < 0.05) than that seen for PL (see Figure 4). Although BET appeared to perform more repetitions at T3 than PL, these differences were not statistically different (p = 0.06). The number of repetitions performed at 90% or greater of peak power in the squat exercise was significantly greater for BET at both T2 and T3 than PL (see Figure 5a), while the number of repetitions performed at 90% or greater of mean power was significant greater for BET than PL at T3 only (Figure 5b).

Table 1 provides the power performance data for the Wingate anaerobic power test, and the vertical jump and bench press throw assessments. Results for the two Wingate trials per testing session were averaged. No significant differences between the groups were seen in peak power, mean power, rate of fatigue and total work. In addition, no significant differences between the groups were seen in either vertical jump power or power performance in the bench press throw at any time point.

**Table 1:** Wingate Anaerobic Power Test, Vertical Jump and Bench Press Throw Power Performance

	**Group**	**T1**	**T2**	**T3**
**WAnT Peak Power (W)**	**PL**	1001 ± 107	1038 ± 128	1034 ± 116
	**BET**	957 ± 184	980 ± 161	958 ± 170
**WAnT Mean Power (W)**	**PL**	609 ± 42	608 ± 38	620 ± 32
	**BET**	592 ± 61	589 ± 41	593 ± 59
**WAnT Rate of Fatigue (w·sec^-1^)**	**PL**	23.2 ± 4.8	24.6 ± 6.0	23.9 ± 5.7
	**BET**	23.9 ± 7.9	24.0 ± 7.2	24.5 ± 8.1
**WAnT Total Work (J)**	**PL**	18270 ± 1266	18245 ± 1152	18605 ± 964
	**BET**	17776 ± 1822	17680 ± 1231	17675 ± 1771
**Vertical Jump Power (W)**	**PL**	4695 ± 754	4617 ± 524	4666 ± 994
	**BET**	4487 ± 1061	4662 ± 1606	4635 ± 1493
**Bench Press Throw Peak Power (W)**	**PL**	514.6 ± 80.8	531.5 ± 77.3	528.4 ± 82.5
	**BET**	547.5 ± 160.2	541.7 ± 156.0	537.0 ± 162.5
**Bench Press Throw Mean Power (W)**	**PL**	317.8 ± 50.4	318.3 ± 47.9	316.7 ± 49.4
	**BET**	331.9 ± 101.2	332.3 ± 99.9	328.5 ± 02.3

All data are reported as Mean ± SD. WAnT = Wingate Anaerobic Power test; PL = Placebo; BET = Betaine

Comparisons between groups in the average profile of mood states scores and soreness ratings can be observed in Table 2. No significant differences were seen between the groups at any time point for any of the mood states. No differences between the groups were seen in soreness ratings as well.

**Table 2:** Profile of Mood States and Soreness Ratings

	**Group**	**T1**	**T2**	**T3**
**Tension**	**PL**	39.7 ± 7.4	38.8 ± 2.1	36.3 ± 2.3
	**BET**	42.5 ± 4.8	39.6 ± 5.6	38.2 ± 6.8
**Depression**	**PL**	37.9 ± 2.1	37.8 ± 2.1	37.1 ± 1.0
	**BET**	37.9 ± 1.6	37.5 ± 1.2	37.7 ± 2.2
**Anger**	**PL**	38.3 ± 1.8	37.8 ± 2.9	38.2 ± 2.1
	**BET**	38.8 ± 3.1	39.5 ± 3.9	39.8 ± 5.5
**Vigor**	**PL**	44.3 ± 11.6	44.2 ± 11.9	40.4 ± 10.8
	**BET**	46.3 ± 6.4	39.4 ± 10.4	37.9 ± 10.1
**Fatigue**	**PL**	41.2 ± 5.5	39.3 ± 3.9	30.6 ± 7.0
	**BET**	42.5 ± 6.5	40.4 ± 6.3	41.5 ± 4.2
**Confusion**	**PL**	36.0 ± 3.6	33.8 ± 3.6	31.7 ± 2.0
	**BET**	35.6 ± 4.5	35.7 ± 7.2	32.3 ± 2.2
**Soreness Ratings**	**PL**	3.9 ± 2.5	4.3 ± 2.2	3.6 ± 2.1
	**BET**	3.7 ± 2.7	2.9 ± 2.2	5.2 ± 2.3

All data are reported as Mean ± SD. PL = Placebo; BET = Betaine

## Discussion

The results of this study indicates that two weeks of betaine ingestion can significantly improve muscle endurance in a lower body workout by increasing the number of repetitions performed in the squat exercise, as well as improve the quality of the workout by improving the number of repetitions performed at 90% of the subject's maximal mean and peak power outputs. These improvements appear to occur within one week of supplementation. This effect was not seen in the upper body measure or in other measures of anaerobic power (Wingate test, vertical jump test or bench press throw).

The results of this study do not support the improved power performance reported by Maresh and colleagues [13]. In addition, the greater number of repetitions performed for the squat exercise in this study also contrasts with the results of that study. The differences between these studies are not clear. Considering that both studies used recreationally trained individuals, it is possible that variability in resistance training experience and jump performance ability seen in this type of subject populations [14], contributed to these differing yet positive results.

The mechanism that is likely contributing to the improved muscle endurance seen in this study is probably related to an increase in muscle creatine concentrations. However, this is only speculative since muscle creatine concentrations were not measured in this study. Other studies have reported that betaine supplementation can increase muscle creatine concentrations, albeit in chickens [16]. No studies are known that have examined changes in muscle creatine concentrations in humans supplementing with betaine. The donation of methyl groups from betaine is thought to occur via a series of enzymatic reactions in the mitochondria of liver and kidney cells [17]. Betaine donates a methyl group to homocysteine to form methionine. This transfer is controlled by the enzyme betaine homocysteine methyl transferase that results in betaine being converted to dimethylglycine. Methionine is converted to S-adenosylmethionine (SAM) which acts as a methyl donor contributing to the synthesis of creatine, as well as number of other proteins [2]. Dietary betaine has been shown to increase serum methionine, transmethylation rate and methionine oxidation in healthy men [18], and animals injected with betaine have shown a dose response increase in red blood cell SAM [19]. However, the relationship of betaine ingestion and muscle creatine synthesis in humans has not been established.

The improved muscle endurance and the greater quality of repetitions (as reflected by a significantly greater number of repetitions performed at 90% of subject's 1-RM) in the squat exercise seen in subjects supplementing with betaine is consistent with benefits typically seen in subjects ingesting creatine [20,21]. Interestingly, significant improvements were realized even after 7-days of supplementation, similar to what one may expect following a loading dose of creatine [22]. However, these ergogenic effects were only seen in the squat exercise and not the bench press exercise. It is possible that the larger muscle mass exercise may have been affected to a greater extent from betaine supplementation than the smaller upper body musculature, or that the experience level of these subjects may have been more focused on upper body training than lower body squat exercises. Previous studies from our laboratory have indicated that performance gains in the squat exercise are often greater in magnitude than that seen in the bench press exercise [23,24]. This has been suggested to be related to the commonality of the bench press exercise in the initial training program of both competitive and recreational athletes, and the inconsistent use of the squat exercise or poor technique (e.g. lowering to parallel position) used in that exercise during training sessions.

The inability to see improvements in power performance from two weeks of betaine supplementation contrasts with results reported by Maresh and colleagues [13]. However, improvements in power performance are often dependent upon these exercises being part of the subjects training program. Similar to previous research examining creatine supplementation, if the specific exercises used to assess power improvements are not part of the subjects training program the ability to see performance improvements may be compromised [20]. This appears to have occurred in this study in that the power exercises were only performed during the testing sessions. Although subjects were expected to still maintain their normal resistance training program during the two-week study, the training program of these subjects did not include bench press throws, plyometric exercises or the Wingate anaerobic power test.

Previous research has suggested that betaine supplementation may enhance mood in a clinical population suffering from motor neuron disease [25]. The results of this study do not provide any support for improved mood or reduction in soreness ratings from two-weeks of betaine ingestion. Additional research needs to be conducted to further explore the potential benefits of betaine on mood.

In conclusion, two-weeks of betaine supplementation in active, college males appeared to improve muscle endurance of the squat exercise, and increase the quality of repetitions performed (e.g. number of repetitions performed at 90% of 1-RM). These performance improvements were realized within 7-days of supplementation. However, no changes in power performance were seen during this study. Additional research is warranted to determine the rate of muscle creatine synthesis from betaine supplementation, and to compare muscle creatine synthesis kinetics from creatine supplementation versus betaine supplementation.

## Competing interests

Danisco-USA, (Ardsley, NY) provided funding for this project. All researchers involved collected, analyzed, and interpreted the results from this study and have no financial interests concerning the outcome of this investigation. Publication of these findings should not be viewed as endorsement by the investigators, The College of New Jersey or the editorial board of the Journal of International Society of Sports Nutrition.

## Authors' contributions

JRH was the primary investigator, obtained grant funds for project, designed study, supervised all study recruitment, data/specimen analysis, statistical analysis and manuscript preparation. NAR, JK, SLR and ADF were co-authors, oversaw all aspects of study including recruitment, data/specimen analysis, and manuscript preparation.
